# Foundations of a life support equipment exchange platform

**DOI:** 10.1051/ject/2023001

**Published:** 2023-03-24

**Authors:** Justin R. Sleasman, Ula Hijawi, Abdullah Alsalemi, Mohamed Rabie, Mohammad Noorizadeh, Aidan Stead, Christopher Cooley, Conor Donnelly, Jonathan W. Haft, Darryl Abrams, Christine Stead, Kathleen R. Ryan, Peter Rycus, Alexander D. Fox, Mark T. Ogino, Peta M.A. Alexander

**Affiliations:** 1 Lead Perfusionist, Stanford Medicine Children’s Health 725 Welch Road Palo Alto CA 94304 USA; 2 Researcher and Project Team Leader, Qatar University Doha Qatar; 3 Interns, Extracorporeal Life Support Organization Ann Arbor MI 48103 USA; 4 Department of Cardiac Surgery, University of Michigan Ann Arbor MI 48109 USA; 5 Division of Pulmonary and Critical Care Medicine, Columbia University Irving Medical Center New York NY 10032 USA; 6 CEO, Extracorporeal Life Support Organization and Adjunct Faculty, University of Michigan School of Public Health, Health Management and Policy Ann Arbor MI 48103 USA; 7 Department of Pediatrics, Division of Cardiology, Stanford Medicine Children’s Health and Stanford University School of Medicine Palo Alto CA 94304 USA; 8 Executive Director, Extracorporeal Life Support Organization Ann Arbor MI 48103 USA; 9 Project Manager, Extracorporeal Life support Organization Ann Arbor MI 48103 USA; 10 Pediatric Neonatal-Perinatal, Critical Care Service, Division of Neonatology Nemours/Alfred I. du Pont Hospital for Children Wilmington DE 19803 USA; 11 Department of Cardiology, Boston Children’s Hospital and Department of Pediatrics Harvard Medical School Boston MA 02115 USA

**Keywords:** Critical Care, Extracorporeal Membrane Oxygenation (ECMO), Supply Chain, COVID-19, ELSO Supply Platform, Pilot Program

## Abstract

*Background*: The Extracorporeal Life Support Organization Supplies Platform (https://Supplies.ELSO.org) was created out of Extracorporeal Membrane Oxygenation (ECMO) disposable product shortage prior to and during the Coronavirus Disease 2019 (COVID-19) pandemic. This novel Platform supports Centers in obtaining disposables from other Centers when alternative avenues are exhausted. *Methods*: Driven by the opportunity for increased patient care by using the product availability of the 962 ELSO centers worldwide was the motivation to form an efficient online supply sharing Platform. The pandemic created by COVID-19 became a catalyst to further recognize the magnitude of the supply disruption on a global scale, impacting allocations and guidelines for institutions, practice, and patient care. *Conclusions*: Records kept on the Platform website are helpful to the industry by providing insights into where difficulties exist in the supply chain for needed equipment. Yet, the common thread is awareness, of how critical situations can stretch resources and challenge our resolve for the best patient care. ELSO is proud to support member centers in these situations, by providing a means of attaining needed ECMO life support products to cover supply shortages.

The Extracorporeal Life Support Organization (ELSO) is an international, non-profit consortium of institutions dedicated to the development and use of therapies for the support of failing organ systems. Extracorporeal Membrane Oxygenation (ECMO) is an invasive, resource-intensive rescue strategy for critically ill patients with respiratory and/or cardiorespiratory failure not responsive to standard therapies [1]. ECMO utilization reported to the ELSO Registry has increased exponentially in the last 10 years [2]. Coupled with the rapid rise in use, manufacturing supply disruptions of cardiopulmonary products on the global market cause intermittent shortages of products that may not be distributed uniformly across centers [3–5]. The COVID-19 pandemic catalyzed supply disruption on a global scale, impacting allocations and availability of ECMO circuit components for patient care [6–8]. The ELSO Supplies Platform (https://Supplies.ELSO.org) was developed to mitigate current and future supply shortage crises.

This novel Platform supports ELSO member centers in acquiring ECMO circuit components (disposables) when standard supply chains and regional sharing arrangements are exhausted. These pragmatic solutions most often occurred in emergency situations, but as the pandemic evolved in 2020, these crises became more frequent. One problem became quite apparent: there was not an efficient system to facilitate product demand. The opportunity for access to disposables required for patient care by leveraging the product availability of the 962 ELSO centers worldwide was the motivation to form an efficient online supply sharing Platform.

The concept of a centralized supply-sharing Platform was raised early in the COVID-19 pandemic at a meeting of professionals at the Seminar of the American Academy of Cardiovascular Perfusion. The ELSO Executive Team and Board of Directors agreed to contribute resources and recruited researchers at Qatar University to build a user-friendly online platform to access.

ECMO disposables when usual supply chains have failed. The online Platform evolved to support the concept of swapping, donating, or borrowing ECMO disposables, starting with membrane oxygenators, since these were the rate-limiting component in ECMO support at the time. The ELSO Supplies team created the unique structure of the Platform, completing it within nine months from conceptual to functional prototype.

With the development of the ELSO Supplies Platform underway, members of the ELSO Steering Committee undertook a survey of member centers regarding perceptions of ECMO disposable limitations prior to and during the COVID-19 pandemic. The survey was pilot tested with ten international center directors, before being optimized and distributed to 723 member centers in July-September 2020. Responses were received from 322 (44.5% response rate) international centers (Figure 1). In brief, 35.4% of responding center directors reported issues with access to ECMO circuit components, either pre-pandemic, during the initial stages of the pandemic, or both pre and during the early pandemic.

**Figure 1:**
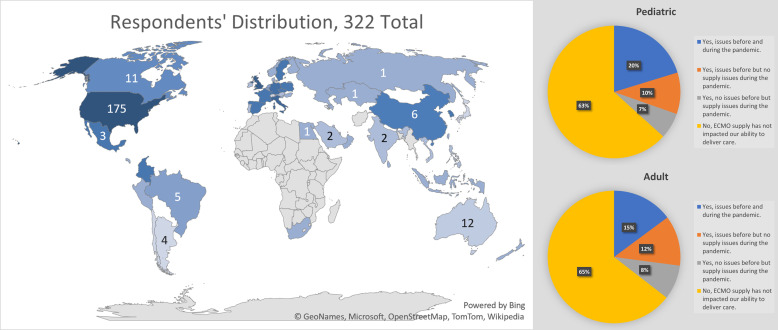
The international distribution of the ELSO Survey Respondents (July–September 2020), and proportion of Pediatric and Adult centers reporting issues with supply of ECMO circuit disposables.

After developing a prototype in 2020, a pilot program to beta test the Platform was established with nine regional centers located in Washington, Oregon, and northern California (USA). These institutions represented both pediatric and adult ELSO Centers with varied ECMO volumes. The beta test was carried out to carefully review and test every step of the supply exchange process in terms of creating a request, processing a request, dispatching equipment, and the underlying logistics. Feedback from the participants of the nine ELSO centers was collected and evaluated as to ease of use and information clarity (Figures 2 and 3).

**Figure 2:**
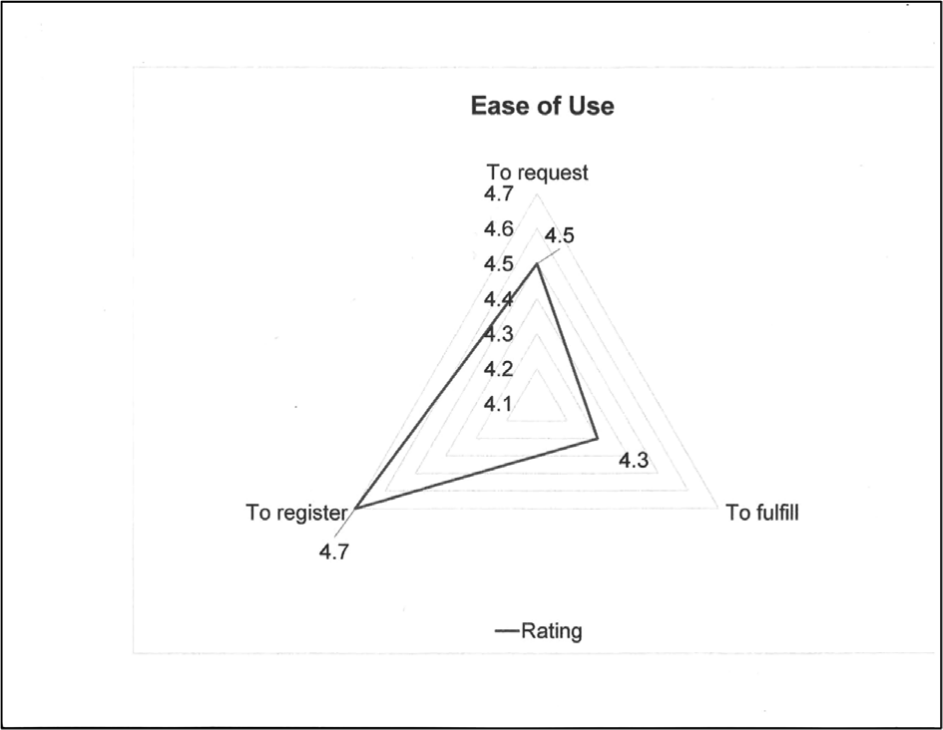
Information clarity on the home page, product page, and request checkout page is rated (out of 5) as 4.7, 4.8, and 4.4, respectively.

**Figure 3:**
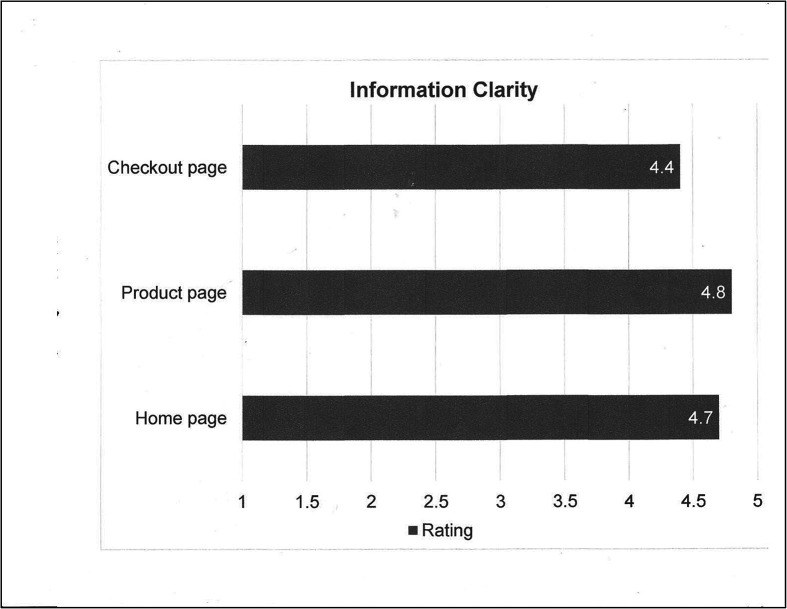
The average response (out of 5) on the ease of use when registering for the platform, requesting equipment, and fulfilling requests are 4.7, 4.5, and 4.3, respectively.

Centers could nominate as “Requestors” (i.e., requesting supplies) and/or “Suppliers” (i.e., fulfilling requests created by Requestors). The Platform assumes the storage of transaction records between users. As a request is entered on the Platform, a notification is sent to all registered centers. Once a supplier fulfills a request, the request is noted as completed on the Platform (Figure 4). In a swap, the request would remain active until the requesting center has sent the product back to the initial supplier. To fulfill a request, shipping between centers was required (Figure 5). FedEx^®^ was established as the domestic shipping company for the Platform, with the shipping application integrated into the Platform interface. The all-in-one system facilitates shipping label printing when supplying a request. Future work includes incorporating informational resources and component standards on equipment listing pages.

**Figure 4:**
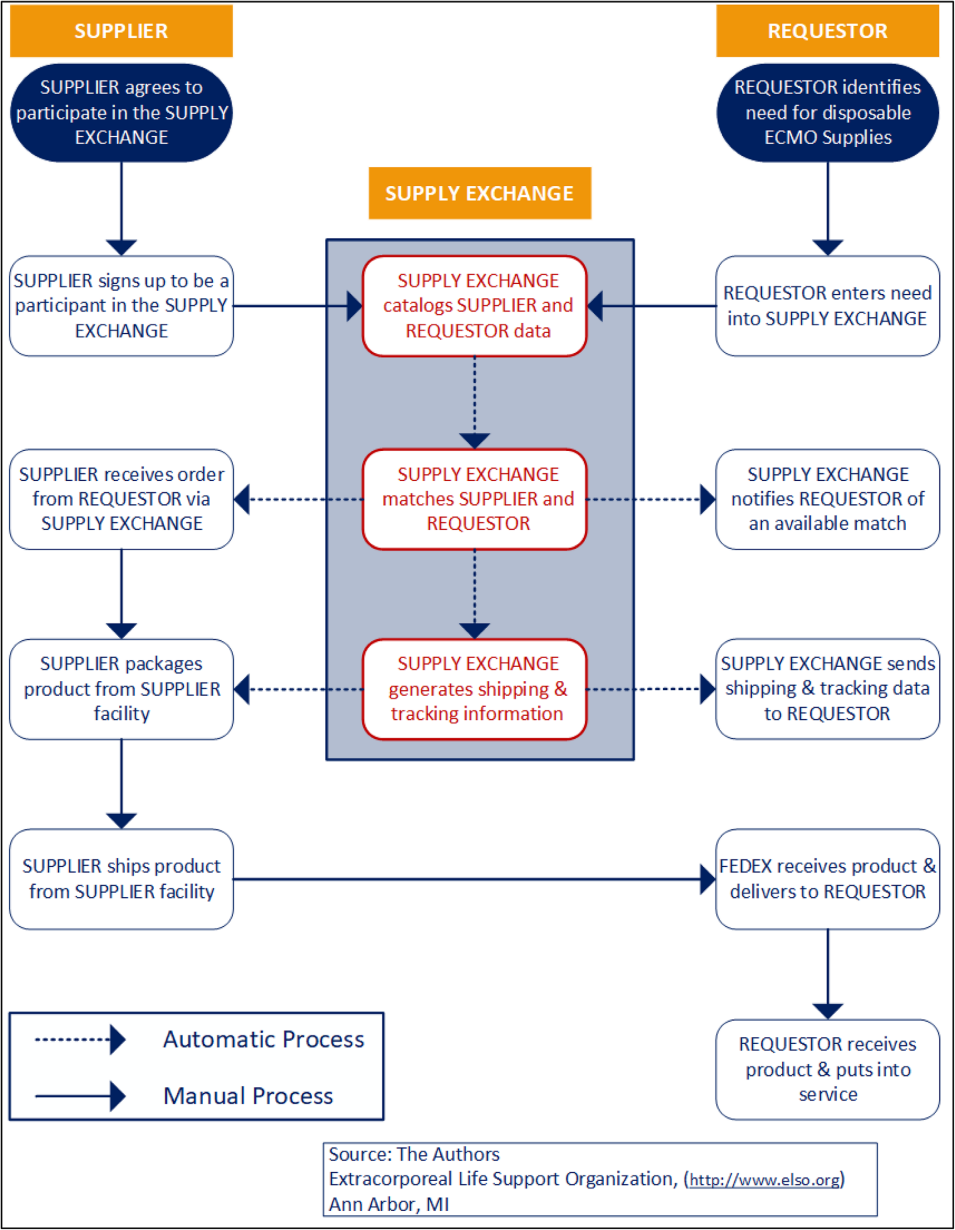
The Extracorporeal Life Support Organization (ELSO) supply exchange process workflow.

**Figure 5:**
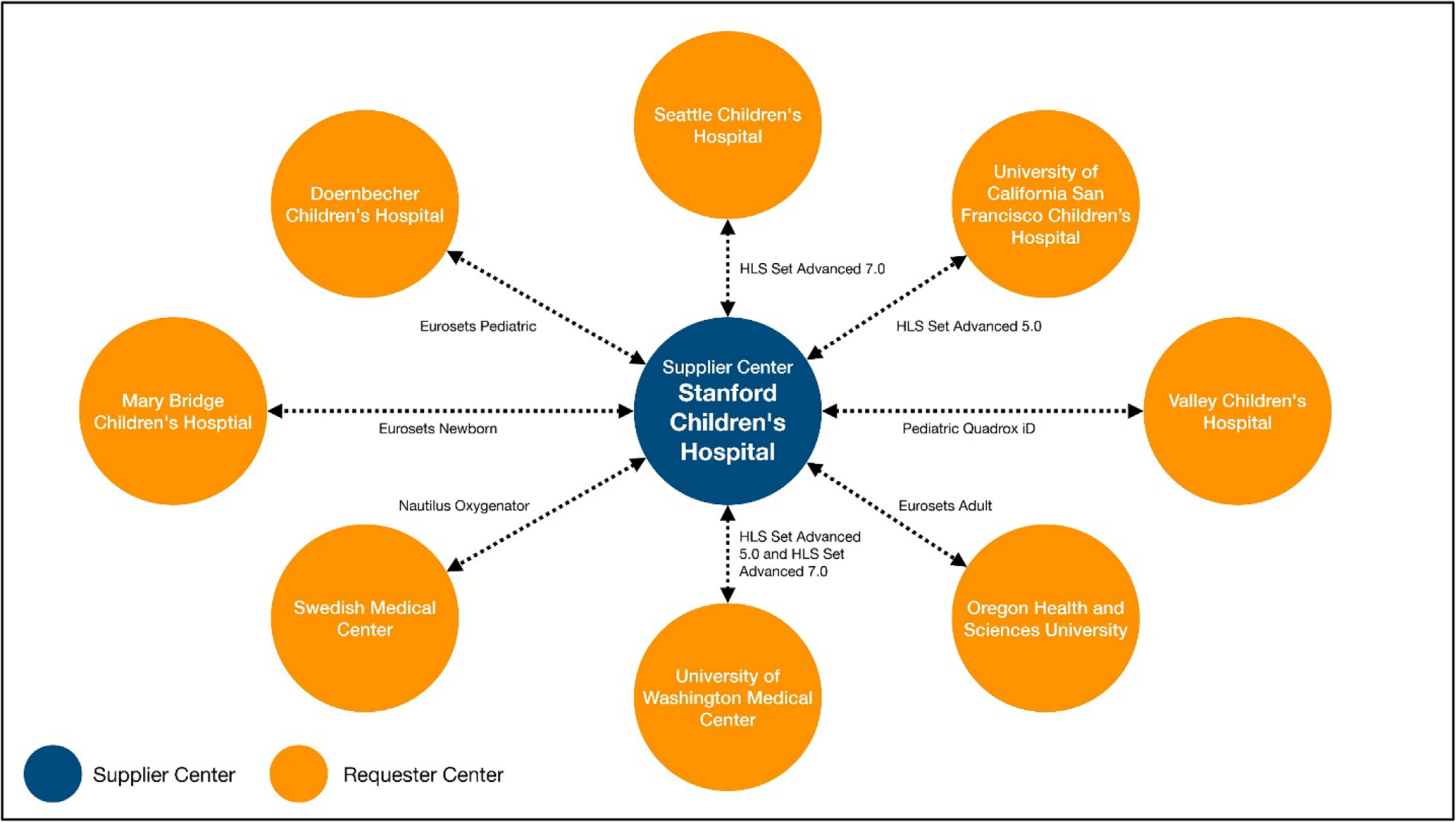
The request fulfillment distributions between requestors and suppliers.

Liability is an area of concern. Therefore, a memorandum of understanding (MOU) was formulated to communicate the mutually accepted expectations of all parties involved in an agreement between the Requestor and Supplier, while protecting ELSO as the moderator. A User Agreement was added to the website as a pop-up document requiring both Requestor and Supplier to complete to become a registered center, with a two-step verification of acceptance [9]. The first step verifies that the participant read the document and the second denotes acceptance of the terms of the agreement. Initially piloted regionally, the Platform expanded nationally (USA), and is projected to evolve globally.

The increased utilization of ECMO circuit components in response to the COVID-19 pandemic led to a crisis in supply chain access that drove novel perspectives and methodologies for problem-solving and creating solutions. ELSO Supplies Platform is one such solution created to address a current and future emergency need when usual supply chains and regional arrangements have failed. Post-pandemic, the Platform will be aimed at a future failsafe for ECMO Equipment emergencies that systematically allows centers to seamlessly share supplies.

## Data Availability

All available data are incorporated into the article.
